# Second generation DNA methylation age predicts cognitive change in midlife: the moderating role of childhood socioeconomic status

**DOI:** 10.18632/aging.206284

**Published:** 2025-07-23

**Authors:** Sophie A. Bell, Christopher R. Beam, Ebrahim Zandi, Alyssa Kam, Emily Andrews, Jonathan Becker, Deborah Finkel, Deborah W. Davis, Eric Turkheimer

**Affiliations:** 1 Department of Psychology, University of Virginia, Charlottesville, VA 22904, USA; 2 Department of Psychology, University of Southern California, Los Angeles, CA 90089, USA; 3 Davis School of Gerontology, University of Southern California, Los Angeles, CA 90089, USA; 4 Keck School of Medicine, University of Southern California, Los Angeles, CA 90089, USA; 5 Department of Family and Geriatric Medicine, University of Louisville School of Medicine, Louisville, KY 40202, USA; 6 Institute for Gerontology, Jönköping University, Jönköping, Sweden; 7 Center for Economic and Social Research, University of Southern California, Los Angeles, CA 90089, USA; 8 Department of Pediatrics, University of Louisville School of Medicine, Louisville, KY 40202, USA; 9 Norton Children’s Research Institute Affiliated with The University of Louisville School of Medicine, Louisville, KY 40202, USA

**Keywords:** epigenetic age, cognition, socioeconomic status, DNA methylation, lifespan

## Abstract

DNA methylation age (DNAmAge) surpasses chronological age in its ability to predict age-related morbidities and mortality. This study analyzed data from 287 middle-aged twins in the Louisville Twin Study (mean age 51.9 years ± 7.03) to investigate the effect of DNAmAge acceleration on change in IQ (ΔIQ) between childhood and midlife, while testing childhood socioeconomic status (SES) as a moderator of the relationship. DNAmAge was estimated with five commonly used algorithms, or epigenetic clocks (Horvath, Horvath Skin and Blood, GrimAge, and PhenoAge). A factor analysis of these measures produced a two-factor structure which we identified as first generation and second generation measures. Results of genetically informed, quasi-causal regression models indicated that accelerated second generation DNAmAge predicted more negative ΔIQ from childhood to midlife, after accounting for genetic and environmental confounds shared by twins. The relationship between DNAmAge and ΔIQ was moderated by childhood SES, with a stronger effect observed among twins from low SES backgrounds. Second generation DNAmAge measures trained to estimate phenotypic biological age show promise in their predictive value for cognitive decline in midlife. Our genetically informed twin design suggested that epigenetic aging may represent a pathway through which early-life socioeconomic disadvantage impacts midlife cognitive health.

## INTRODUCTION

Aging is highly heterogeneous, and chronological age is not a sensitive measure of the unique physiological and developmental processes that occur across the human lifespan. Patterns of DNA methylation (DNAm) across the genome can be used to estimate DNA methylation age (DNAmAge), a family of measures strongly correlated with chronological age but better able to predict morbidities and mortality. Adjusting for chronological age, DNAmAge measures can be residualized to generate measures of acceleration [1], such that positive residuals reflect faster biological aging, while negative residuals reflect slower, and presumably healthier, aging. Since the advent of the first measures a decade ago, DNAmAge measures reliably correlate with demographic characteristics like education level and socioeconomic status (SES) as well as clinically relevant outcomes including cognitive performance in adulthood [2, 3].

### Predicting cognitive outcomes from DNAmAge

Individuals with accelerated DNAmAge have an increased risk of cancer, stroke, heart disease, and neurodegenerative disease [4–6]. While cognitive functioning is a crucial facet of both healthy and pathological aging, the link between DNAmAge and cognitive functioning remains unclear, partly due to inconsistencies in the predictive accuracy of various DNAmAge algorithms [7]. First generation DNAmAge measures, such as Horvath, Horvath Skin and Blood, and Hannum DNAmAge, were designed primarily to estimate chronological age with methylation data from cytosine-phosphate-guanine (CpG) sites sensitive to time-dependent changes [1, 8, 9]. While two studies found that Horvath DNAmAge was associated with cognitive decline [6, 10], several others have found no statistically significant associations between first generation DNAmAge measures and cognitive performance or decline [11–14].

In contrast, second generation DNAmAge measures, including PhenoAge and GrimAge, are trained to predict age-related outcomes by integrating DNAm signatures tied to clinical biomarkers, health behaviors (e.g., smoking), and demographic variables [15, 16]. These algorithms aim to capture phenotypic aging processes beyond chronological age, such as morbidity and mortality risk. Accelerated PhenoAge and GrimAge are consistently associated with lower cognitive performance in middle and older adulthood [11, 12, 17, 18]. However, findings on cognitive decline are mixed. Neither Hillary et al. [18] nor Maddock et al. [12] reported statistically significant results, yet Reed and colleagues [13] found that those with cognitive decline in midlife showed more accelerated GrimAge scores than those without decline. These findings suggest that second generation measures, developed to more accurately estimate phenotypic biological age, may better index age-related changes in cognitive ability.

### Early-life exposures and pace of DNAmAge

Disadvantaged socioeconomic status (SES) in childhood is an established risk factor for lower cognitive ability across the lifespan and late-life cognitive decline [19]. Recent research has also begun exploring links between childhood adversity and later-life DNA methylation age (DNAmAge). In retrospective accounts of adversity (e.g., poverty, death of parent, alcohol/drug use in family, abuse), only poverty was related to GrimAge acceleration [20]. Childhood SES but not adult SES predicted accelerated DNAmAge in midlife using the Horvath and Hannum clocks, suggesting the methylome, or DNA methylation modifications across the genome, may be particularly vulnerable to external stressors in childhood [21, 22]. However, findings remain inconsistent: McCrory et al. [23] found no association between childhood SES and Horvath, Hannum, or PhenoAge DNAmAge in adults over 50, and unexpectedly, Faul et al. [11] found that low childhood SES was associated with slower aging on the Horvath and Hannum clocks later in life. It is not yet known whether SES moderates the effects of DNAmAge on cognitive outcomes.

### Genetics, epigenetics, and classical twin designs

Associations between early life exposures, mid- to late-life outcomes, and DNAmAge have primarily been correlational. Two studies decomposed the variance of DNAmAge into additive genetic, shared environmental, and nonshared environmental components using a traditional twin model [24, 25], while two others used pedigree designs to estimate heritability [16, 26]. Our group has shown how to use a longitudinal twin design to go beyond estimating heritability while sharpening causal inference between a predictor and outcome [27]. Only two studies have examined the relationship between DNAmAge and cognitive ability while controlling for between-family confounds [10, 14]. In the Middle-Aged Danish Twin Study, no relationship was observed between the Horvath and Hannum clocks and cognitive change over 10 years in midlife [14]. Vaccarino and colleagues [10] found no associations between Horvath, Hannum, GrimAge, or PhenoAge clocks and baseline cognition in middle-aged male twins. However, there was a statistically significant within-twin pair effect of Horvath DNAmAge on cognitive decline 11 years later. The reason why only Horvath’s original measure predicted cognitive ability is unclear, as initial validation studies of PhenoAge and GrimAge reported associations with cognitive outcomes [15, 16].

Using the Louisville Twin Study (LTS), we expanded on this limited amount of research examining DNAmAge and cognitive change and sought to clarify how early life environmental characteristics may influence midlife cognitive outcomes. The LTS, initiated in 1957, includes intensive, prospective data on twins’ development from infancy through adolescence and recent re-evaluations in midlife on their cognitive, physical, and psychosocial functioning as well as epigenetic aging [28]. Parental data collected during the twins’ childhood further enabled us to examine how early life exposures, here rearing SES, relate to twins’ midlife epigenetic and cognitive aging.

The current study used a twin design to investigate causal effects of DNAmAge on change in IQ (ΔIQ) between childhood and midlife, while testing childhood SES as a moderator of the relationship. While observational designs cannot reproduce the causal inference possible with random assignment, twins offer a quasi-experimental method for studying causal processes while controlling for family-level genetic and environmental confounds. If a monozygotic (MZ) twin has a higher DNAmAge and also a greater ΔIQ than their co-twin, the association cannot be due to genetic differences or early environmental exposures, because identical twins reared together are matched for these factors. For this reason, we refer to associations that survive twin-based controls as “quasi-causal” [27]. To address variability in DNAmAge algorithms, we conducted an exploratory factor analysis of five widely used measures. We expected second generation DNAmAge measures to be sensitive to ΔIQ in midlife, as they were developed to capture phenotypic aging processes beyond chronological age. Finally, given recent findings linking early life stressors with midlife DNAmAge, we hypothesized that the relationship between midlife DNAmAge and ΔIQ would depend on differences in childhood SES.

## RESULTS

### Descriptive statistics

The sample includes 287 individual twins, comprising 60 monozygotic and 41 dizygotic complete pairs and 85 single twins. All participated in the childhood and midlife phases of the LTS (Supplementary Figure 1). Twins were assessed in person using three versions of the Wechsler Intelligence Scale for Children (WISC) and the Wechsler Adult Intelligence Scale (WAIS-IV). We used Wechsler Full-Scale Intelligence Quotient (IQ) scores as an overall estimate of an individual’s level of global cognitive ability. The average age for childhood IQ was 14.42 (1.93) years, and the average age for adult IQ was 51.87 (7.03). Mean childhood IQ was 102.7 (13.4), and mean adult IQ was 105.4 (13.8) (Table 1). DNAmAge was estimated using principal components (PC) based epigenetic clock methodology which has shown significantly improved reliability and stability over time as compared with the traditional Horvath, Horvath Skin and Blood, Hannum, PhenoAge, and GrimAge algorithms [29]. After regressing DNAmAge values on chronological age and adjusting for cell type, Horvath, Horvath Skin and Blood, Hannum, PhenoAge, and GrimAge had means close to zero (Table 1). MZ twin correlations (0.60-0.74) were approximately double those for DZ twins (0.28-0.37) across most measures, except PhenoAge, where MZ and DZ correlations were more similar (0.56 vs. 0.47). Pairwise clock correlations are in Supplementary Table 1.

**Table 1:** Sample characteristics of Louisville Twins.

**Characteristic**	**Total n (%)**	**Mean (SD)**	**MZ correlation^1^** **r (SE)**	**DZ correlation^2^** **r (SE)**
***Sociodemographic factors***	287			
Age, years		51.87 (7.03)		
Age for childhood IQ, years		14.42 (1.93)		
Female Sex	169 (58.89)			
White	262 (91.29)			
Non-Hispanic	279 (99.29)			
Education, 16 or more years	161 (56.09)			
Childhood SES^3^		46.09 (26.02)		
Smoker	66 (23.00)			
***Cognitive function***				
Childhood IQ Score	282	102.67 (13.44)	.88 (.06)	.58 (.14)
Adult IQ Score	255	105.45 (13.84)	.87 (.07)	.50 (.14)
***DNAmAge^4^***	281			
Horvath		0.00 (3.37)	.62 (.11)	.37 (.15)
Horvath Skin and Blood		-0.03 (3.59)	.62 (.10)	.32 (.15)
Hannum		-0.01 (3.25)	.60 (.11)	.28 (.16)
PhenoAge		-0.09 (4.20)	.56 (.11)	.47 (.14)
GrimAge		-0.12 (3.42)	.74 (.09)	.37 (.15)
Gen 1		0.00 (2.85)	.62 (.10)	.32 (.15)
Gen 2		0.00 (1.78)	.71 (.09)	.42 (.15)

^1^Monozygotic Twin Pair Correlation.

^2^Dizygotic Twin Pair Correlation.

^3^Childhood socioeconomic status (SES) measured using the Duncan Socioeconomic Index.

^4^DNAmAge is controlled for chronological age and cell composition. Residuals are in years.

Results from standardized univariate twin models decomposing the variance of individual DNAmAge measures showed little to no shared environmental variance, C, in Horvath, Horvath Skin and Blood, Hannum, and GrimAge clocks. Because C was estimated at a negative value in four of five ACE models initially, C was set to zero, and the constrained AE models are presented (Supplementary Table 2). The five DNAmAge measures were substantially heritable, with the additive genetic effects, A, ranging from 57% to 73%, and non-shared environment, E, accounting for 27% to 43% of the variance in DNAmAge (Supplementary Table 2).

### Exploratory factor analysis of DNAmAge measures

We conducted an exploratory factor analysis (EFA) using the five DNAmAge measures residualized for age and cell count. Based on Eigenvalues and fit indices, we selected a two-factor model (RMSEA = 0.08; 90% CI: [0.00, 0.20]) (Supplementary Figure 2). Results from a likelihood ratio test comparing a two-factor model to a one-factor model suggested that the two-factor model fit substantially better (χ^2^ =70.07, df=4, p < .001). First generation DNAmAge measures (Horvath, Horvath Skin and Blood, and Hannum) loaded on one factor, while second generation measures (PhenoAge and GrimAge) loaded on a second factor. We refer to these factors as “Gen 1” and “Gen 2”, respectively. Factor loadings (p < .05) are displayed in Supplementary Table 3. The factors were correlated *r* = 0.57. Composite scores were generated for each participant based on the EFA. Gen 1 is the sum of standardized values for Horvath, Horvath Skin and Blood, and Hannum DNAmAge (M=0.00, SD= 2.85). Gen 2 is the sum of standardized values for GrimAge and PhenoAge DNAmAge (M=0.00, SD= 1.78). Twin pair correlations for Gen 1 and Gen 2 are in Table 1.

### Univariate twin models of Gen 1 and Gen 2 DNAmAge

We decomposed the variances of Gen 1 and Gen 2 into A, C, and E components using the classical twin model. Both Gen 1 and Gen 2 were moderately heritable, with A accounting for 50% of the variance in Gen 1 and 60% of the variance in Gen 2. The variance attributable to the non-shared environment was 42% in Gen 1 and 31% in Gen 2, while shared environment accounted for less than 1% of the variance in both. Standardized estimates are in Supplementary Table 2.

### Phenotypic and “quasi-causal” association models

**Figure 1:**
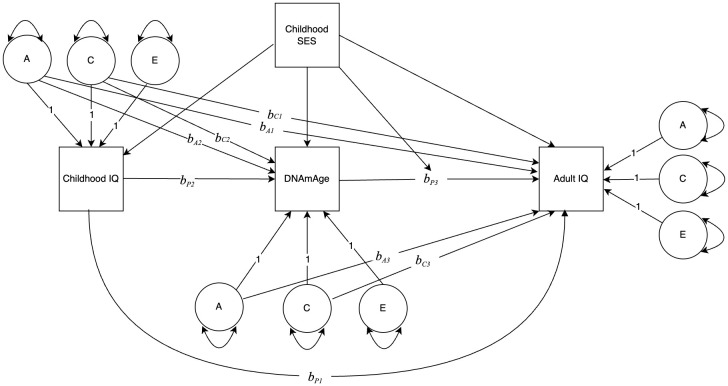
**Path diagram depicting the association between DNAmAge and change in IQ, modified by childhood socioeconomic status (SES).** Variances of ACE components are estimated, with paths to observed variables set to 1.0. All regression coefficients are unstandardized. *b_A1_* and *b_C1_* are regression coefficients from the A and C components of Childhood IQ to Adult IQ. *b*_P1_ is the phenotypic regression conditional on *b_A1_* and *b_C1_*. *b_A2_* and *b_C2_* are regression coefficients from the A and C components of Childhood IQ to DNAmAge. *b_P2_* is the phenotypic regression conditional on *b_A2_* and *b_C2_*. *b_A3_* and *b_C3_* are regression coefficients from the A and C components of DNAmAge to Adult IQ. *b_P3_* is the phenotypic regression conditional on *b_A3_* and *b_C3_*. SES is a covariate for DNAmAge and the IQ variables, and a moderator of *b_P3_*.

Following the twin modeling framework outlined by Turkheimer and Harden [27], we first estimated a “phenotypic association” model with no control for differential regressions between and within twin pairs. We then fit a “quasi-causal” regression model, using the ACE decomposition of the predictor variable to control for family-level genetic and environmental confounds in the individual-level regressions. This sequence (phenotypic association model and quasi-causal regression model) was estimated for Gen 1 then repeated for Gen 2. Figure 1 illustrates the model: adult IQ was regressed on childhood IQ, DNAmAge, and the A and C components of each; DNAmAge was regressed on childhood IQ and its A and C components; SES (a family-level variable) was included as a linear covariate for DNAmAge and IQ variables and as an interaction term with DNAmAge. By including childhood IQ in the model, the conditional effects of SES and DNAmAge can be interpreted as predicting change in IQ from childhood to midlife (ΔIQ). The phenotypic regression coefficient (*b*_P3_) is an estimate of the relationship between the DNAmAge and ΔIQ with between-family genetic and environmental confounds controlled, i.e., within a pair of identical twins reared together.

### Predictors of DNAmAge

Phenotypic models. In the phenotypic association model, there were no statistically significant associations with Gen 1 DNAmAge (Supplementary Table 4). However, accelerated Gen 2 DNAmAge was associated with lower childhood SES (*b=*-0.41, SE=0.13, p<.05) (Table 2).

**Table 2:** Phenotypic and quasi-causal regression model results for Gen 2 DNAmAge.

**Gen 2 DNAmAge**						
		**Childhood IQ** **estimate (SE)**		**DNAmAge** **estimate (SE)**		**Adult IQ** **estimate (SE)**
**Phenotypic Association Model**	**SES**					
	***b***	0.56 (0.09)**	***b***	-0.67 (0.20)**	***b***	0.20 (0.11)
	**Childhood IQ**					
		_	***b_A2_***	_	***b_A1_***	_
		_	***b_C2_***	_	***b_C1_***	_
		_	***b_P2_***	-0.08 (0.09)	***b_P1_***	0.48 (0.14)**
	**DNAmAge**					
		_		_	***b*_A3_**	_
		_		_	***b*_C3_**	_
		_		_	***b_P3_***	-0.10 (0.03)*
	**DNAmAge X SES**					
					***b_int_***	0.06 (0.03)*
	**Variances**					
	**A**	1.15 (0.34)		1.96 (0.31)		0.50 (0.08)
	**C**	0.15 (0.32)		0.00 (0.00)^†^		0.00 (0.00)^†^
	**E**	0.22 (0.04)		0.85 (0.16)		0.24 (0.05)
**Quasi-Causal Regression Model**	**SES**					
	***b***	0.56 (0.09)**	***b***	-0.67 (0.19)*	***b***	0.18 (0.12)
	**Childhood IQ**					
		_	***b_A2_***	-0.78 (0.41)	***b_A1_***	0.16 (0.23)
		_	***b_C2_***	0.30 (1.42)	***b_C1_***	0.80 (0.67)
		_	***b_P2_***	0.38 (0.26)	***b_P1_***	0.48 (0.14)**
	**DNAmAge**					
		_		_	***b*_A3_**	0.13 (0.15)
		_		_	***b*_C3_**	0.00 (0.00)
		_		_	***b_P3_***	-0.18 (0.07)*
	**DNAmAge X SES**					
					***b_int_***	0.07 (0.03)*
	**Variances**					
	**A**	1.06 (0.29)		1.73 (0.56)		0.43 (0.12)
	**C**	0.21 (0.28)		0.00 (0.00)^†^		0.00 (0.00)^†^
	**E**	0.23 (0.04)		0.80 (0.15)		0.23 (0.05)

b_P_, phenotypic association between predictor and outcome; b_A_, amount of variance attributable to additive genetic influences; b_C_, amount of variance attributable to shared environmental experiences; b_int_, estimate of the interaction effect. Data are presented as unstandardized regression coefficients with standard errors in parentheses.

^†^C variances have been set to zero.

*p <.05 **p≤ .001.

Quasi-causal models. Next, we used the quasi-causal regression model to estimate the effects of childhood IQ and SES on DNAmAge, using the ACE decomposition of childhood IQ to control for genetic and environmental family-level confounds. There were similarly no statistically significant predictors of Gen 1 DNAmAge. In the Gen 2 quasi-causal analyses, childhood SES again predicted Gen 2 DNAmAge (*b=*-0.67, SE=0.19, p<.001), while childhood IQ did not predict Gen 2 DNAmAge (Table 2). Genetic and environmental confounds were not significantly different from zero.

### Predictors of midlife cognitive functioning

Phenotypic models. In the phenotypic association models for both Gen 1 and Gen 2, childhood IQ statistically predicted adult IQ. The strong relationship between childhood IQ and adult IQ (*r* = 0.77) is also illustrated in Supplementary Figure 3, where adult IQ is regressed on childhood IQ without controlling for the differential regressions between and within twin pairs. Similarly, SES was positively associated with childhood IQ (*b*=0.56, SE=0.09, p <.001) in both phenotypic association models. Higher childhood SES predicted higher adult IQ in the Gen 1 model (*b*=0.16, SE=0.07, p<.05), though this association did not meet statistical significance in the Gen 2 model (Supplementary Table 4). Gen 1 and ΔIQ were not associated with each other (Supplementary Table 4). We did find a statistically significant phenotypic association between Gen 2 and ΔIQ (*b*_P3_= -0.10, SE = 0 .03, *p* <.05). In both Gen 1 and Gen 2 phenotypic association models, we then tested the interaction between DNAmAge and SES (*b*_int_) in the prediction of ΔIQ but found that the interaction was not statistically significant.

Quasi-causal models. In quasi-causal models, we then regressed adult IQ on childhood IQ (*b*_P1_), DNAmAge (*b*_P3_), and the A and C components of both variables. There was a statistically significant phenotypic association between childhood IQ and adult IQ after accounting for genetic and environmental confounds shared by identical twins in both Gen 1 (*b_P1_* = 0.41, p <.01) and Gen 2 models (*b_P1_* = 0.48, p <.001). This association shows that over 40% of the variance in adult IQ can be attributed to childhood IQ. Consistent with the phenotypic association results above, accelerated Gen 2 DNAmAge predicted more negative ΔIQ from childhood to midlife (*b* = −0.18, SE=0.07, *p* <.05). Genetic and environmental confounds (*b*_A3_ and *b*_C3_) were not significantly different from zero. The quasi-causal twin model implies that within a pair of MZ twins raised together, the member of the pair with accelerated DNAmAge also declines more in cognitive ability between childhood and midlife. These findings were only apparent when DNAmAge was measured with Gen 2 DNAmAge.

**Figure 2:**
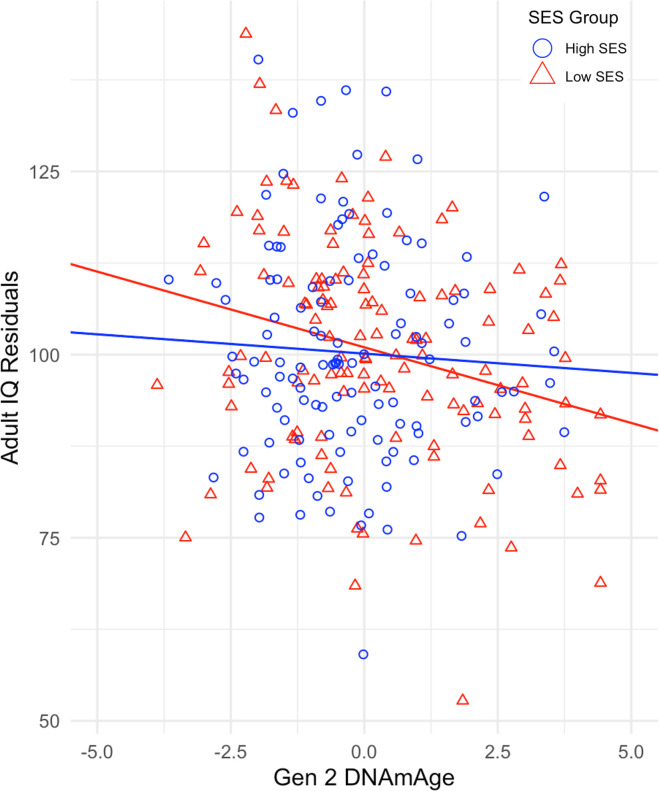
**Regression of Adult IQ residuals on Gen 2 DNAmAge for high and low SES groups.** IQ residuals are derived from the regression of adult IQ on childhood IQ and childhood socioeconomic status.

We then tested the interaction between DNAmAge and SES (*b*_int_) in the quasi-causal models. The interaction revealed that twins raised in lower SES families showed a more negative relationship between Gen 2 DNAmAge and ΔIQ (*b* = 0.07, SE=0.03, *p* <.05) (Table 2). Figure 2 illustrates the moderating effect of childhood SES by using a median split of our sample for SES and displaying the regression of adult IQ on Gen 2 DNAmAge in both high- and low-SES groups. The effect of DNAmAge on ΔIQ is greater in twins raised in lower SES households, indicating that socioeconomic disadvantage may be an early exposure that amplifies the negative effects of epigenetic aging on cognitive trajectories in midlife.

Given smoking is both a risk factor for cognitive decline and is also correlated with types of DNA methylation, we repeated our quasi-causal regression models testing smoking as a covariate. The GrimAge clock, in particular, should be the most sensitive to smoking as it was trained on a DNAm-based surrogate of smoking pack years [16, 30], so we expected smoking to be significantly related to Gen 2 DNAmAge. In fact, smoking was strongly associated with Gen 2 DNAmAge (*b* = 1.98, SE=0.22, *p* <.001). With smoking included as a covariate, and one degree of freedom removed, the association between Gen 2 and adult IQ was no longer statistically significant; however, the effect was in the same direction (*b* = −0.12, SE=0.07, *p* =.09). The interaction effect was similarly no longer statistically significant but was in the same direction (Supplementary Table 5).

## DISCUSSION

DNAmAge is an effective predictor of specific aspects of biological aging, but challenges remain due to the lack of a gold standard DNAmAge measure and variations in the predictive accuracies of various measures. Most studies have focused on associations between DNAmAge and phenotypic aging outcomes, but few have tested whether such associations occur because DNAm causes biological aging as opposed to merely correlating with it. Our study first identified two factors across five DNAmAge measures and then used a longitudinal twin design to improve causal inference between early-life exposures, DNAmAge, and ΔIQ in midlife.

As hypothesized, Gen 2 DNAmAge, but not Gen 1, predicted ΔIQ in midlife. This result was consistent with several previous studies showing associations between second generation measures and cognition cross-sectionally [11, 17] and longitudinally [13]. PhenoAge and GrimAge algorithms both incorporate methylation signatures of clinical markers—PhenoAge from blood-based biomarkers predicting physical function, multimorbidity, and mortality [15], and GrimAge from plasma proteins and smoking pack years selected for their prediction of time-to-death [16]. These second generation DNAmAge measures have already shown improvements over first generation DNAmAge in predicting physical functioning, morbidity, and lifespan [16, 31]. Our finding that accelerated Gen 2 DNAmAge predicted more negative ΔIQ suggests that decline in cognitive ability is interwoven with other age-related physiological processes that associate with the methylome. Given that our main effect of Gen 2 DNAmAge on ΔIQ was no longer statistically significant when smoking was included as a covariate, we cannot fully disentangle the effects of DNAmAge and smoking on cognitive decline in this analysis. A larger dataset is needed to parse the two. Recent work has shown that GrimAge is one pathway through which life course smoke exposure predicts chronic physical health conditions (e.g., cancer, lung disease) and mortality in adulthood [30]. Our results build on this, suggesting that DNAmAge measures that incorporate smoking may also act as useful biomarkers of cognitive health status.

The within-twin pair association between Gen 2 DNAmAge and ΔIQ that withstood controls for between family level confounds suggests that differences in environmental exposures partially explain their association. Two prior twin studies of DNAmAge and cognition had mixed findings [10, 14]. Our Gen 1 findings were consistent with Starnawska and colleagues [14] who did not find within-twin pair associations between Horvath and Hannum DNAmAge and cognition cross-sectionally or longitudinally. Our results differed from Vaccarino et al. [10] who found no within-pair association between PhenoAge and GrimAge and cognitive decline. Differences in study design may explain this discrepancy: while our sample included both male and female twins, Vaccarino et al. examined only male veterans. Our study also spanned a longer age range, predicting change from childhood and adolescent IQ to midlife, whereas Vaccarino et al. used two testing points within participants’ 50s and 60s. Importantly, the LTS lifespan longitudinal design allowed us to capture change from premorbid assessments of cognitive ability to midlife, a period when subtle, age-related declines may emerge.

While some prior studies found that lower childhood SES was associated with accelerated DNAmAge on the Horvath and Hannum clocks [21, 22], others detected no significant effect [23, 32]. Our results supported the hypothesis that early-life SES would differentially affect Gen 1 and Gen 2 DNAmAge. Childhood SES predicted Gen 2 DNAmAge but not Gen 1 DNAmAge in midlife, consistent with findings from McCrory et al. [20], who found childhood poverty was associated with accelerated GrimAge in 50- to 87-year-olds. Childhood poverty has been found to influence health across the lifespan by limiting access to resources like education and healthcare [33]. However, the interaction effect between DNAmAge and SES in our study offers a novel explanation for one pathway through which early-life stressors become biologically embedded. The relationship between accelerated epigenetic age and cognitive decline was amplified within twin pairs from lower-income households, suggesting that those raised in poorer families are more vulnerable to the cognitive effects associated with the broader physiological aging measured by DNAmAge.

Using the classical twin model, we estimated the genetic, shared environmental, and nonshared environmental variance components of five DNAmAge measures, Gen 1, and Gen 2. Consistent with the small number of previous twin studies estimating DNAmAge heritability, we found that the variance in DNAmAge measures was best explained by the AE model, with C constrained to zero [24, 25]. Encouragingly, our heritability estimates for PC-trained Horvath, Hannum, PhenoAge, and GrimAge were mostly consistent with those reported by Hong and colleagues in a large sample of middle-aged Chinese twins [25].

To our knowledge, only one previous study has conducted a factor analysis on multiple DNAmAge measures. In a model predicting multimorbidity and activities of daily living, Faul and colleagues [11] found Horvath and Hannum measures loaded on a first factor and PhenoAge, GrimAge, and DunedinPACE loaded on a second factor. Our EFA results aligned with this, identifying a “Gen 1” factor with Horvath, Horvath Skin and Blood, and Hannum clocks, and a “Gen 2” factor with PhenoAge and GrimAge. Although we did not calculate DunedinPACE, a “third generation” measure that estimates rate of decline over nearly twenty years, future studies should compare its sensitivity with Gen 2 in the LTS sample [34]. These results contribute to an understanding of the shared underlying patterns of measurement in “Gen 1” and “Gen 2” DNAmAge. Distinguishing between these two domains is a step towards refining the application of DNAmAge: our findings suggest second generation DNAmAge measures may have greater utility in studies of cognitive aging than first generation DNAmAge.

### Strengths and limitations

Unlike previous studies that relied on retrospective reports of childhood financial hardship or parental occupation, ours is the first to use a prospective rating of childhood SES, which may have reduced recall bias and improved reliability. However, our childhood SES measure was a relatively crude indicator of parental occupation, and incorporating additional socioeconomic factors could strengthen future research. We did not test adult SES in this study, given that previous work had suggested childhood SES, but not adult SES, was predictive of DNAmAge [21, 22]. While our sample covered a broad socioeconomic range, it was predominantly white and recruited from one region of the United States, limiting generalizability. Future studies should examine the relationship between second generation DNAmAge measures and patterns of cognitive ability in larger and more diverse samples. Finally, we used IQ score as a proxy for global cognitive ability and future work should examine relationships between DNAmAge and specific cognitive domains including memory and executive functioning.

## MATERIALS AND METHODS

### Participants

Participants were 287 individual twins from the midlife phase of the Louisville Twin Study (LTS). The LTS began as a study of childhood development in multiple birth pairs. Twins born between 1950-1997 in the Louisville metropolitan area were followed and assessed on cognitive ability, physical development, and temperament between 3 months and 15 years of age. Parents of twins provided data on demographics, parenting factors, and home environment. Zygosity was determined through blood typing during childhood. The sample contains same-sex monozygotic (MZ) and dizygotic (DZ) twin pairs, and opposite-sex DZ pairs. The LTS closed down in 2000 after 1,770 individual twins had been recruited [35]. In 2018, follow-up of LTS twins aged 30-65 was initiated [28], and LTS reopened in 2019 to begin the first midlife data collection of the 1,770 twins. Participants provided a 50cc whole blood sample for genotyping and DNA methylation assay. Genotyping confirmed zygosity for any unknown twin pairs. The present sample includes 60 MZ pairs, 41 DZ pairs, and 85 individual twins who completed a midlife study visit between 2019-2023. Singletons were included in the analysis and contributed to between-pair analyses but not to within-pair regressions. Supplementary Figure 1 illustrates the participant flow, attrition, and retention across study phases.

### Socioeconomic status

Childhood socioeconomic status (SES) was measured using the Duncan socioeconomic index which assigns scores of 0-100 based on the occupation of the head of the household [36]. Occupational prestige was based on 1950 US Census income data and education level associated with the occupation. The childhood SES of our sample (M=47.3, SD = 25.7) was consistent with the overall LTS distribution (M=46.9, SD= 26.9), evenly distributed, and representative of the Louisville, Kentucky area [35].

### Smoking

During the midlife assessment, participants self-reported whether they had smoked tobacco in the last three years. We coded responses at 1 (smoked in the last three years and/or current smoker) and 0 (has not smoked in the last three years).

### Cognitive ability measures

LTS participants were assessed using validated Wechsler batteries of cognitive functioning. Children and adolescents were administered one of three forms of the Wechsler Intelligence Scale for Children (WISC, WISC-R, WISC-III) [37–39]. Adults in the midlife phase were assessed using the Wechsler Adult Intelligence Scale IV (WAIS-IV) [40]. Test scores are age-standardized based on a standardization sample that is roughly representative of the United States. Wechsler IQ scores have a mean of 100 and a standard deviation of 15. Children were typically administered cognitive assessments at ages 7, 8, 9, 12, and 15, though not all twins were assessed at each timepoint; this study used the most recently available WISC assessment for each participant, with over 80% collected at age 15 (see Table 1 for more detailed descriptive statistics).

### DNAmAge measures

Genomic DNA was extracted from whole-blood samples that were collected at the University of Louisville and Norton Healthcare medical campuses. Venous blood was collected in ethylenediaminetetraacetic acid (EDTA) tubes and shipped to the Norris Comprehensive Cancer Institute at the University of Southern California Keck School of Medicine. DNA was extracted with promega Maxwell 16 LEV blood DNA kits, and then treated with bisulfite reagents at the USC Molecular Genomics Core following manufacturer protocol. Methylation was assayed with the Illumina Infinium Human MethylationEPIC BeadChip (Illumina, San Diego, CA, USA) at the USC Molecular Genomics Core at the Norris Cancer Institute at the Keck School of Medicine.

Quality control was conducted in R 4.3.0 [41] using the minfi package [42] to identify aberrant samples and CpG sites, remove cross-reactive probes, conduct background correction, and adjust for batch effects prior to estimating DNAmAge variables. Background correction was performed using the normal-exponential out-of-band (noob) method [43]. The ComBat method was used to adjust for laboratory batches [44].

We estimated blood cell composition using the Houseman method [45] and obtained percentages of CD8+ T cells, CD4+ T cells, natural killer cells (NK), B cells, monocytes, and granulocytes. Cell proportions are highly correlated, so in the current analysis we included the first and second principal components from a principal components (PC) analysis (collectively, they explained 86.12% of the variance) in all analyses.

As replicate blood samples often have different methylation values due to noise in the CpG sites, batch effects, and sample preparation, Higgins-Chen et al. [29] developed a principal components-based method to bolster reliability in DNAmAge algorithms by training them based on PC analysis, extracting the covariance between multicollinear CpGs, including age-related covariance. We estimated DNAmAge using five PC-trained algorithms, Horvath, Horvath Skin and Blood, Hannum, PhenoAge, and GrimAge [1, 8, 9, 15, 16].

### Data analysis

Descriptive statistics for the sample’s demographic characteristics, survey data, and cognitive ability data were computed in R version 4.3.2 [41]. Methylation data were cleaned and prepared in R. DNAmAge was calculated as the residual difference in PC-trained values predicted from chronological age in a bivariate linear regression model. DNAmAge values were adjusted for cell composition. Age and cell-adjusted variables were then winsorized such that values more than two standard deviations from the mean DNAmAge score were replaced with a value of two standard deviations above or below the mean. Pairwise correlations for the five DNAmAge measures were then estimated using maximum likelihood in Mplus Version 8.8 [46].

We used the classical twin model to decompose the phenotypic variance of DNAmAge into additive genetic (A), shared environmental (C), and nonshared environmental (E) components. Given that MZ twins share 100% of their genetic makeup and DZ twin pairs share half, on average, the A variance is correlated 1.0 within MZ twin pairs and 0.5 within DZ twin pairs. In both MZ and DZ twin pairs, the C component includes experiences that make siblings raised together more similar, and is correlated 1.0. Nonshared environmental components are unique to individuals and by definition uncorrelated within twin pairs. We estimated a univariate ACE model for all five DNAmAge measures individually using Mplus. Because C values were estimated as negative, we selected to use the constrained AE models, with C set to zero. AE estimates with 95% confidence intervals calculated using bias corrected bootstrap standard errors are presented in Supplementary Table 2.

Exploratory factor analysis was conducted in Mplus to identify underlying dimensions across the five DNAm clock variables (Horvath, Horvath Skin and Blood, Hannum, PhenoAge, and GrimAge). The number of factors to be rotated was determined by the scree plot of Eigenvalues (Supplementary Figure 2) and a likelihood ratio test comparing a two-factor model to a one-factor model. EFA models were estimated using full-information maximum likelihood with an oblique (geomin) rotation. Factor loadings were evaluated for statistical significance, with a criterion of p < .05 (Supplementary Table 3). We estimated composite scores based on the results of the EFA by standardizing the DNAmAge variables and summing the standardized values for the variables that loaded on each factor. As described in the results, the composite scores for each factor are referred to as Gen 1 and Gen 2. Each of the following models was fit for Gen 1 then repeated for Gen 2.

We first estimated univariate ACE models for Gen 1 and Gen 2 DNAmAge, decomposing their variances into genetic, shared environmental, and nonshared environmental components. Next, to explore the primary aims of the study, the relationship between DNAmAge and ΔIQ was modeled in two ways. Both the phenotypic association model and “quasi-causal” regression model were fit in Mplus. The phenotypic association model is equivalent to a simple phenotypic regression where the relationship between predictor and outcome is examined without controlling for genetic and shared environmental effects. This was estimated prior to all genetically informed models. Using a dataset wide by twin pair and estimated parameters set to be equal across the members of a pair, we regressed adult IQ on childhood IQ, childhood SES, DNAmAge, and the interaction between SES and DNAmAge.

Finally, we fit the quasi-causal regression model to estimate the phenotypic regression coefficients conditional on *b*_A_ and *b*_C_ paths. The classical twin method was used to partition childhood IQ, adult IQ, and DNAmAge into A, C, and E components. In this case, adult IQ was regressed on childhood IQ, DNAmAge, and the A and C components of DNAmAge and childhood IQ, as illustrated in Figure 1. Childhood SES was used as a linear covariate for DNAmAge and the IQ variables. We also estimated an interaction between childhood SES and DNAmAge to determine whether the strength of the relationship between DNAmAge and ΔIQ depended on the SES of the family in which twins were raised. A statistically significant *b***_P3_** coefficient affirmatively answers the question: Within a pair of identical twins, does the twin with the higher DNAmAge value also have the larger positive or negative ΔIQ, statistically adjusting for the effects of the A and C components of DNAmAge (*b*_A3_ and *b*_C3_).

## Supplementary Material

Supplementary Figures

Supplementary Tables
