# Supplementary material for: Second generation DNA methylation age predicts cognitive change in midlife: the moderating role of childhood socioeconomic status
Source: Aging (Albany NY). 2025 Jul 23;17(7):1702–20. doi: 10.18632/aging.206284 (PMC12339020; doi:10.18632/aging.206284)
Supplement: Supplementary Figures [file aging-17-206284-s001.pdf]

SUPPLEMENTARY FIGURES

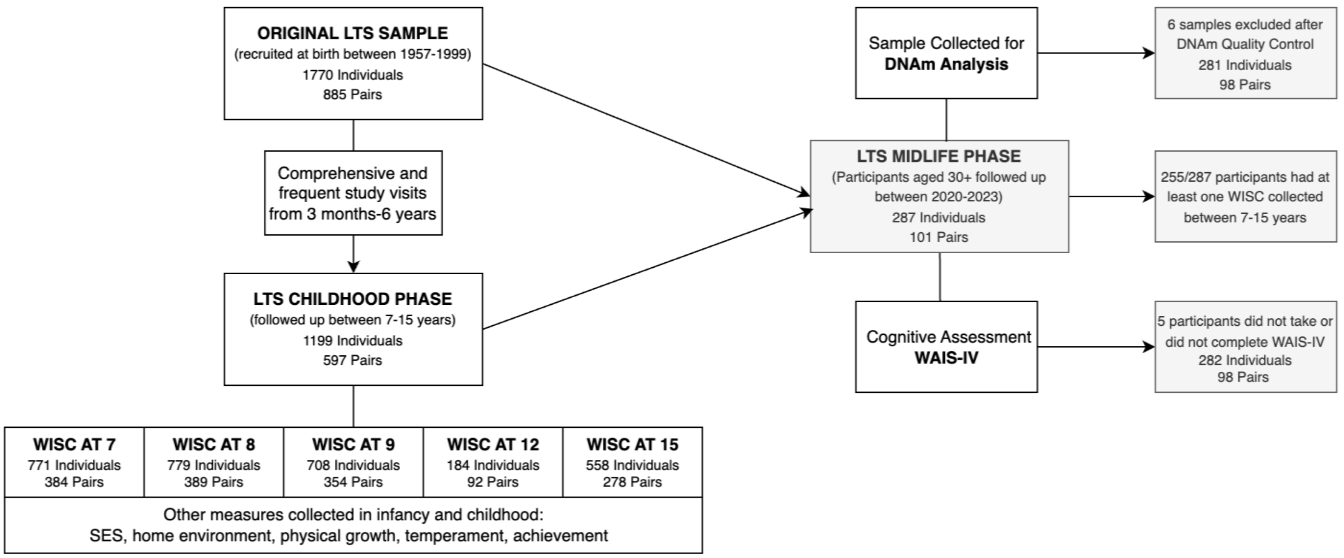

Supplementary Figure 1. Participant flow diagram from the Louisville Twin Study.

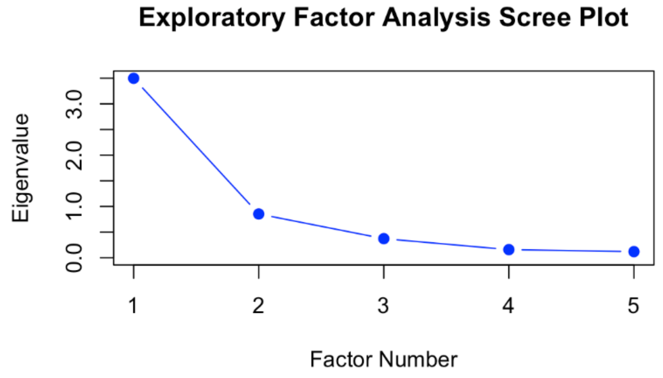

Supplementary Figure 2. Scree plot of eigenvalues.

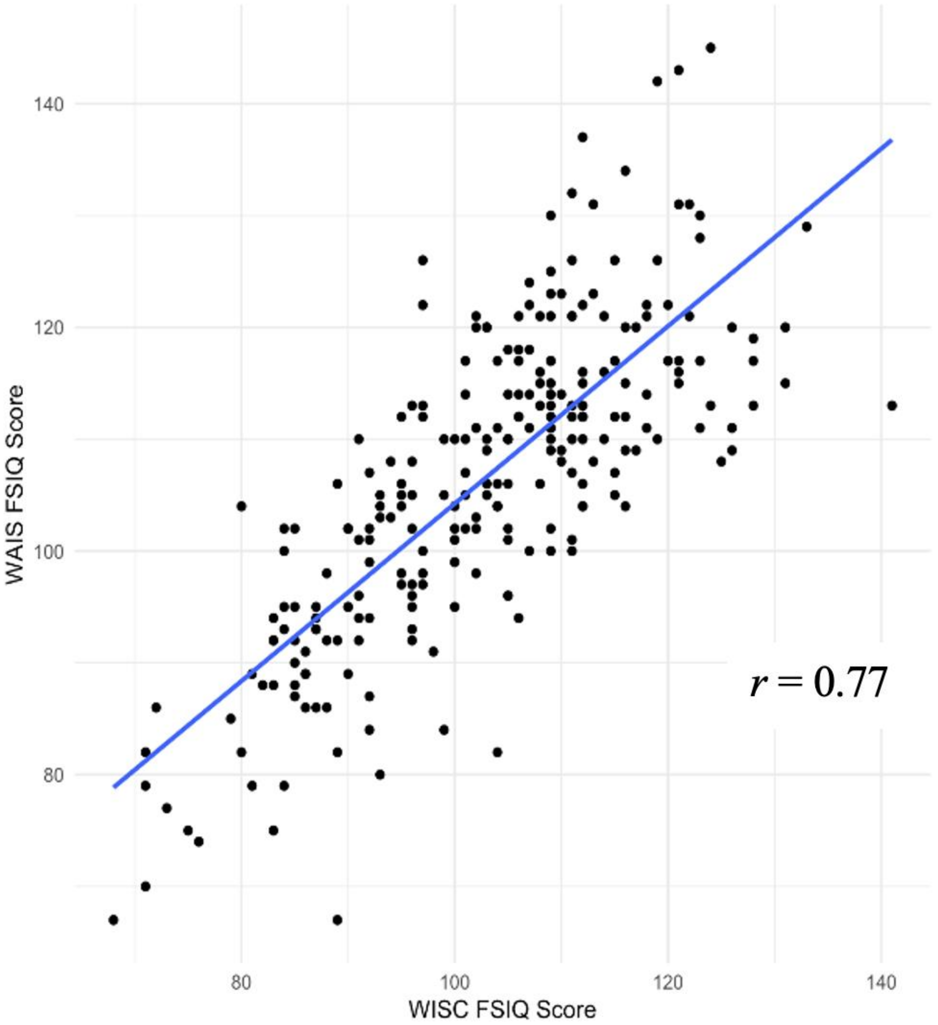

Supplementary Figure 3. Relationship between childhood and adult IQ scores.
