# Supplementary material for: Second generation DNA methylation age predicts cognitive change in midlife: the moderating role of childhood socioeconomic status
Source: Aging (Albany NY). 2025 Jul 23;17(7):1702–20. doi: 10.18632/aging.206284 (PMC12339020; doi:10.18632/aging.206284)
Supplement: Supplementary Tables [file aging-17-206284-s002.pdf]

SUPPLEMENTARY TABLES

Supplementary Table 1. Pairwise correlations between DNAmAge measures.

DNAmAge measures	Horvath	Horvath skin and blood	Hannum	PhenoAge	GrimAge
Horvath	_				
Horvath Skin and Blood	0.86	_			
Hannum	0.87	0.84	_		
PhenoAge	0.57	0.60	0.61	_	
GrimAge	0.37	0.44	0.39	0.58	_

Correlations were estimated after DNAmAge values were adjusted for age and cell composition and were winsorized for values more than two standard deviations above or below the mean. Correlations were computed without regard for twin pair relatedness. All correlations were statistically significant at p<.001.

Supplementary Table 2. Standardized DNAmAge measure ACE decompositions.

	A	C	E
	**Estimate (95% CI)**	**Estimate (95% CI)**	**Estimate (95% CI)**
**Horvath**	0.60 (0.39-0.75)	0.00*	0.40 (0.25-0.61)
**Horvath Skin & Blood**	0.61 (0.39-0.76)	0.00*	0.39 (0.24-0.61)
**Hannum**	0.57 (0.31-0.74)	0.00*	0.43 (0.26-0.69)
**PhenoAge**	0.58 (0.42-0.70)	0.00*	0.42 (0.30-0.58)
**GrimAge**	0.73 (0.55-0.85)	0.00*	0.27 (0.15-0.45)
**Gen 1**	0.50 (-0.24-1.29)	0.08 (-0.65-0.68)	0.42 (0.25-0.67)
**Gen 2**	0.60 (0.10-1.20)	0.09 (-0.50-0.54)	0.31 (0.22-0.44)

A, additive genetic variance; C, shared genetic variance; E, nonshared environmental variance; SE, standard error estimate.
*C is set to zero because variance estimated as negative.

Supplementary Table 3. Results of the exploratory factor analysis.

DNAmAge measure	Gen 1<br>*Loading (SE)*	Gen 2<br>*Loading (SE)*
**Horvath**	**1.00 (0.05)**	-0.10 (0.07)
**Horvath Skin and Blood**	**0.88 (0.03)**	0.06 (0.06)
**Hannum**	**0.92 (0.01)**	0.00 (0.00)
**PhenoAge**	0.30 (0.16)	**0.59 (0.18)**
**GrimAge**	0.00 (0.00)	**0.77 (0.12)**

Bolded values indicate statistical significance at p < .05. The correlation between Gen 1 and Gen 2 is 0.57 (0.12), p < .05.

Supplementary Table 4. Phenotypic and quasi-causal regression model results for Gen 1 DNAmAge.

				Gen 1 DNAmAge		
		**Childhood IQ estimate (SE)**		**DNAmAge estimate (SE)**		**Adult IQ estimate (SE)**
**Phenotypic Association Model**	**SES**					
	b	0.56 (0.09)**	***b***	-0.14 (0.22)	***b***	0.16 (0.07)*
	**Childhood IQ**					
		_	b_{A2}	_	b_{A1}	_
		_	b_{C2}	_	b_{C1}	_
		_	b_{P2}	0.03 (0.15)	b_{P1}	0.71 (0.05)**
	**DNAmAge**					
		_		_	b_{A3}	_
		_		_	b_{C3}	_
		_		_	b_{P3}	-0.01 (0.02)
	**DNAmAge X SES**					
					b_{int}	0.01 (0.02)
	**Variances**					
	**A**	1.15 (0.34)		5.75 (0.93)		0.50 (0.09)
	**C**	0.12 (0.32)		0.00 (0.00)†		0.00 (0.00)†
	**E**	0.22 (0.04)		2.15 (0.45)		0.27 (0.05)
**Quasi-Causal Regression Model**	**SES**					
	b	0.56 (0.09)**	***b***	-0.13 (0.11)	***b***	0.32 (0.11)*
	**Childhood IQ**					
		_	b_{A2}	-0.05 (0.66)	b_{A1}	0.33 (0.23)
		_	b_{C2}	0.36 (1.21)	b_{C1}	0.61 (0.48)
		_	b_{P2}	0.02 (0.45)	b_{P1}	0.41 (0.14)*
	**DNAmAge**					
		_		_	b_{A3}	-0.01 (0.08)
		_		_	b_{C3}	0.00 (0.00)
		_		_	b_{P3}	0.00 (0.05)
	**DNAmAge X SES**					
					b_{int}	0.01 (0.02)
	**Variances**					
	**A**	1.03 (0.29)		5.71 (0.95)		0.46 (0.10)
	**C**	0.22 (0.28)		0.00 (0.00)†		0.00 (0.00)†
	**E**	0.23 (0.04)		2.15 (0.45)		0.26 (0.05)

b_P, phenotypic association between predictor and outcome; b_A, amount of variance attributable to additive genetic influences; b_C, amount of variance attributable to shared environmental experiences; b_{int}, estimate of the interaction effect. Data are presented as unstandardized regression coefficients with standard errors in parentheses.

†C variances have been set to zero.

*p <.05 **p≤ .001.

Supplementary Table 5. Quasi-causal regression model results for Gen 2 DNAmAge with smoking as a covariate.

Gen 2 DNAmAge					
	**Childhood IQ estimate (SE)**		**DNAmAge estimate (SE)**		**Adult IQ estimate (SE)**
**SES**					
b	0.55 (0.09)**	***b***	-0.35 (0.18)*	***b***	0.20 (0.11)
**Childhood IQ**					
	_	b_{A2}	-0.38 (0.44)	b_{A1}	0.20 (0.22)
	_	b_{C2}	0.38 (1.30)	b_{C1}	0.64 (0.53)
	_	b_{P2}	0.15 (0.26)	b_{P1}	0.47 (0.14)**
**DNAmAge**					
	_		_	b_{A3}	-0.02 (0.41)
	_		_	b_{C3}	0.21 (0.25)
	_		_	b_{P3}	-0.12 (0.07)
**DNAmAge X SES**					
				b_{int}	0.05 (0.03)
**Smoking**					
b	-0.06 (0.17)	***b***	1.98 (0.22)**	***b***	-0.21 (0.19)

b_P, phenotypic association between predictor and outcome; b_A, amount of variance attributable to additive genetic influences; b_C, amount of variance attributable to shared environmental experiences; b_{int}, estimate of the interaction effect. Data are presented as unstandardized regression coefficients with standard errors in parentheses.
*p ≤.05 **p≤ .001.
